# Potency of a thermostabilised chimpanzee adenovirus Rift Valley Fever vaccine in cattle

**DOI:** 10.1016/j.vaccine.2016.03.061

**Published:** 2016-04-29

**Authors:** Pawan Dulal, Daniel Wright, Rebecca Ashfield, Adrian V.S. Hill, Bryan Charleston, George M. Warimwe

**Affiliations:** a The Jenner Institute, University of Oxford, Old Road Campus Research Building, Oxford OX3 7DQ, UK; b The Pirbright Institute, Ash Road, Pirbright, Woking GU24 0NF, UK; c Centre for Research on Therapeutic Sciences and, Institute for Healthcare Management Strathmore University, P.O. Box 59857-00200, Nairobi, Kenya

**Keywords:** Thermostable, Adenovirus vaccine, Rift Valley Fever

## Abstract

Development of safe and efficacious vaccines whose potency is unaffected by long-term storage at ambient temperature would obviate major vaccine deployment hurdles and limit wastage associated with breaks in the vaccine cold chain. Here, we evaluated the immunogenicity of a novel chimpanzee adenovirus vectored Rift Valley Fever vaccine (ChAdOx1-GnGc) in cattle, following its thermostabilisation by slow desiccation on glass fiber membranes in the non-reducing sugars trehalose and sucrose. Thermostabilised ChAdOx1-GnGc vaccine stored for 6 months at 25, 37 or 45 °C elicited comparable Rift Valley Fever virus neutralising antibody titres to those elicited by the ‘cold chain’ vaccine (stored at −80 °C throughout) at the same dose, and these were within the range associated with protection against Rift Valley Fever in cattle. The results support the use of sugar-membrane thermostabilised vaccines in target livestock species.

## 1 Introduction

Storage and deployment of vaccines whilst maintaining a cold chain accounts for a major cost of effective human and veterinary immunisation programs. In resource-limited settings, where the burden of vaccine-preventable illness is high and logistical challenges such as lack of uninterrupted electricity supply and poor transport links abound, maintaining the vaccine cold chain is particularly difficult. Safe and efficacious vaccines whose potency is unaffected by long-term storage at ambient temperature would substantially reduce deployment costs, reduce vaccine wastage that occurs following breakdown of the cold chain and potentially improve vaccine coverage [1], [2], [3].

Adenoviruses are among the most promising platforms for development of safe, novel candidate vaccines against human and animal diseases. These non-enveloped double-stranded DNA viruses have been adapted to produce replication-deficient vaccine vectors and evaluated in humans and a wide range of animal species, with excellent safety, immunogenicity and efficacy against many diverse pathogens [4], [5], [6]. However, like many other live vaccine vectors, adenoviruses are heat labile, necessitating cold chain storage of vectored vaccines utilising the platform.

Previously, we developed a thermostabilisation method termed ‘sugar-membrane technology’ for heat labile vaccines that involves their formulation in a stabilising solution of nonreducing disaccharides trehalose and sucrose, followed by drying onto fibrous membranes at ambient temperature to form an inert sugar-glass that thinly coats the fibrous membrane [7]. The impregnated membranes allow storage of vaccine for long periods of time, with very little loss of active material following reconstitution with liquid buffer. Using this technology, a replication-deficient adenovirus vaccine encoding a malaria antigen was previously stored at ambient temperature or 37 °C for 15 months and at 45 °C for up to 6 months, without significant loss in viral titre or immunogenicity [7]. However, this proof-of-concept study was performed in a mouse model and not in the target species for the disease (i.e. humans).

Here, we determined whether a sugar-membrane thermostabilised adenovirus vaccine could be deployed in an actual vaccine target population. Our target disease was Rift Valley Fever, a mosquito-borne viral zoonosis endemic in Africa and the Arabian Peninsula caused by an enveloped negative-stranded RNA virus [8]. The disease occurs as recurrent epizootics of febrile illness in ruminants, with very high mortality rates in young sheep, goats and cattle, and abortion in pregnant animals. Contact with animal tissues or body fluids contaminated with Rift Valley Fever virus (RVFV) is a major route of infection for humans, in whom disease primarily occurs as a self-limiting febrile illness that occasionally progresses to severe manifestations associated with high (>30%) case fatality rates or debilitating sequelae [9], [10], [11]. No licensed vaccines are currently available for humans and the live RVFV virus vaccines widely used for livestock in Africa have major drawbacks, including residual virulence, need for high containment during production, and variable immunogenicity [12]. Furthermore, though available as lyophilised products, the bioactivity of these live RVFV livestock vaccines still relies on a cold chain [12], [13].

To address these issues, we previously developed ChAdOx1-GnGc, a replication-deficient chimpanzee adenovirus vaccine encoding the RVFV envelope glycoproteins that are major targets of a protective neutralising antibody response [14], [15]. Unlike the whole RVFV livestock vaccines in current use [12], [16], ChAdOx1-GnGc contains only the protective immune targets of RVFV making its use compatible with readily available kits that distinguish infected from vaccinated animals on the basis of seropositivity for other RVFV antigens. This is a key consideration for effective disease control during outbreaks [12]. Single-dose immunisation with ChAdOx1-GnGc is highly immunogenic and provides 100% protection against RVFV challenge in sheep, goats and cattle [15], making it a promising candidate for deployment in livestock and humans. Thus, we here evaluated the potency of thermostabilised ChAdOx1-GnGc vaccine in cattle and compared this with the ‘cold-chain’ version of vaccine used in these prior studies.

## 2 Materials and methods

ChAdOx1-GnGc was prepared by Gateway® recombination between the ChAdOx1 destination vector and an entry plasmid containing the coding sequence for RVFV envelope glycoproteins (Genbank accession number DQ380208, bases 411–3614) as described [14]. Standard methods were used for viral rescue, propagation in HEK293 cells and subsequent purification by CsCl gradient ultracentrifugation. The vaccine stock stored in production buffer (10 mM Tris, pH 7.4) at −80 °C was thawed and pooled together to prepare a working stock. Infectivity titre of the working stock was measured to be 3.15 × 10^10^ infectious units (IU)/ml. For thermostabilisation, aliquots of the working stock were formulated in an unbuffered 0.5 M solution containing a mixture of trehalose and sucrose, pipetted onto Whatman® S14 glass fiber (GF) membranes, and dried in a low relative humidity environment in a drying chamber at ambient temperature [7]. No freeze-drying steps were involved. The dried ChAdOx1-GnGc-loaded GF membranes each contained approximately 5 × 10^8^ IU of ChAdOx1-GnGc vaccine. These were placed in bijou vials (two 1 cm^2^ GF membranes per vial), packaged in heat-sealed moisture barrier bags (Dri-Shield 3000, 3 M) and stored at 25, 37, 45 or 55 °C for 6 months in heat chambers equipped with temperature-monitoring probes. Comparisons of vaccine titre and vaccine-elicited immune responses in cattle were then made between the storage conditions in relation to the control ‘cold chain’ ChAdOx1-GnGc vaccine (i.e. liquid vaccine stored in production buffer at −80 °C). Animal experiments were performed at the Pirbright Institute, UK in accordance with institutional and national Home Office guidelines.

## 3 Results and discussion

**Fig. 1:**
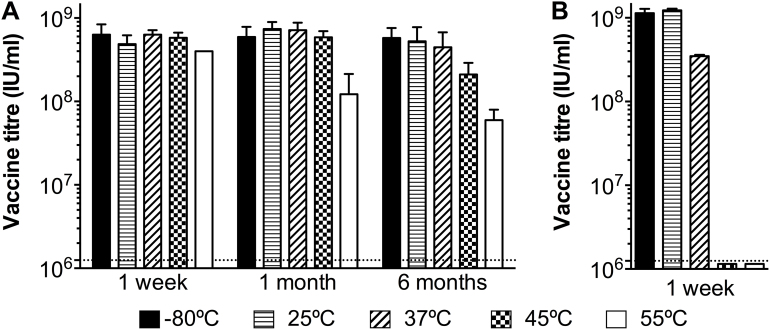
ChAdOx1-GnGc can readily be thermostabilised. In (a) the titre of thermostabilised ChAdOx1-GnGc vaccine following reconstitution in production buffer after storage for 1 week, 1 month or 6 months at the indicated temperatures is shown. In (b), the titre of ‘cold chain’ ChAdOx1-GnGc vaccine after storage for 1 week at the same range of temperatures is shown. Dashed line represents the detection limit.

We first examined the effect of thermostabilisation on vaccine infectivity titre after storage at these temperatures. Thermostabilised ChAdOx1-GnGc vaccine on GF membranes in each sample vial was reconstituted in 500 μl production buffer after storage for 1 week, 1 month and 6 months. The infectious titre of the vaccine was determined by an immunoassay on HEK293 cells as described [17]. Consistent with our previous proof-of-concept study [7], we were able to recover viable vaccine at all storage temperatures and time points (Fig. 1a). In contrast, when storing the ‘cold chain’ ChAdOx1-GnGc vaccine in liquid form for 1 week at the same range of temperatures, recovery of viable vaccine was only possible at 25 and 37 °C, with lower titres at the latter temperature (Fig. 1b). Titres of the thermostabilised ChAdOx1-GnGc vaccine were comparable to the control ‘cold chain’ vaccine (i.e. stored at −80 °C throughout) at the 1-week time point for all temperatures (Fig. 1a). However, marked reduction in viability was observed after storage for 1 month at 55 °C, with a more than tenfold loss in titre observed at 6 months.

Next, we determined the immunogenicity of the thermostable ChAdOx1-GnGc vaccine in cattle, a major target species for a Rift Valley Fever vaccine [12]. The immunogenicity endpoint was induction of RVFV neutralising antibody as this is the main correlate of protection [18], [19]. Three-month old Holstein-Friesian calves were sourced from commercial farms in the UK and randomly allocated into six groups. Groups 1−4 received thermostable ChAdOx1-GnGc vaccine (*n* = 4 per group) reconstituted from the GF membranes in 1 ml production buffer after storage for 6 months at 25, 37, 45 or 55 °C, respectively. Group 5 received the control ‘cold chain’ ChAdOx1-GnGc vaccine (*n* = 4), whereas calves in group 6 (*n* = 2) were left unvaccinated. All vaccinations were intramuscularly administered as a single dose on the right hind limb. Blood for immunological assays was sampled pre-vaccination and at week 4 post-vaccination, after which all animals were culled.

**Fig. 2:**
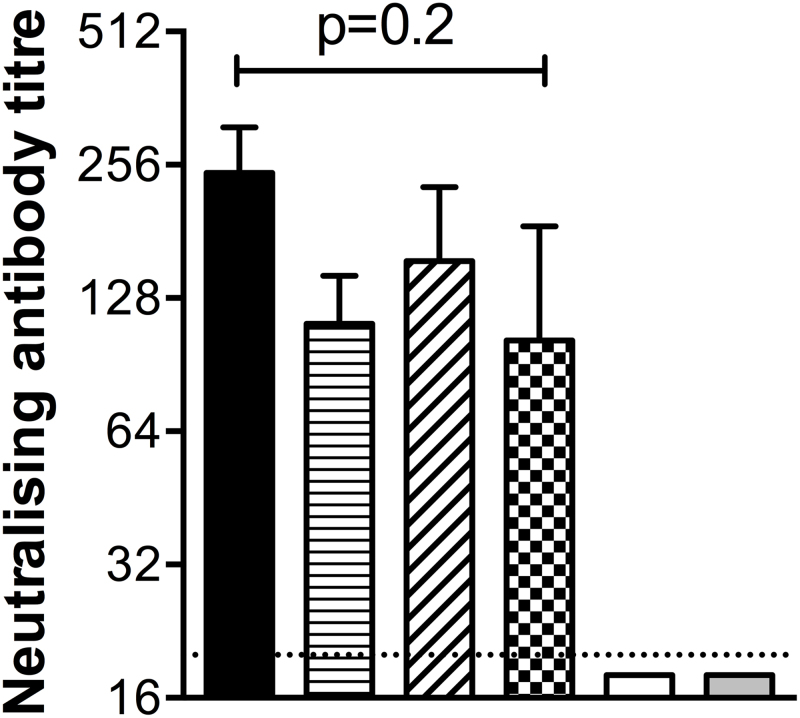
Thermostabilised ChAdOx1-GnGc elicits functional antibody. The titre of RVFV neutralising antibody elicited by vaccination with thermostabilised ChAdOx1-GnGc following 6 months storage at the indicated temperatures is shown. Briefly, sera were heat-inactivated at 56 °C for 30 min and serially diluted in quadruplicate in buffer before incubation with 100TCID_50_ of RVFV MP-12 strain for 60 min at 37 °C. This serum-virus mixture was then transferred onto confluent Vero cell monolayers, incubated at 37 °C and 5% CO_2_ for 72 h, fixed and stained, and endpoint titres calculated by the Spearman-Karber method as described [15]. Data represent mean ± standard deviations. A two-tailed *p*-value representing a between-group comparison by the Kruskal−Wallis test is shown. The 55 °C and unvaccinated (control) groups had no detectable response and are excluded from this analysis.

No local (swelling, pruritus or erythema) or systemic (inappetance, or other clinical signs) adverse events were observed among any of the animals following vaccination. Two calves, one in the ‘cold chain’ vaccine group and the other in the 37 °C group, developed unrelated respiratory illness during the course of follow up and were culled before the end of the study. With the exception of calves receiving vaccine stored at 55 °C, all vaccinees mounted a functional antibody response able to neutralise live RVFV *in vitro* (Fig. 2). Though slightly variable between groups there was no evidence for a statistically significant difference between the neutralising antibody titres elicited by the thermostable and ‘cold chain’ vaccines (Kruskal-Wallis test *p* = 0.2), and the titres were within the range associated with protection against RVFV in cattle (Fig. 2) [15].

Together, the results further support the utility of the sugar-membrane technology in thermostabilising adenovirus-vectored vaccines. Though these viral vectors have been utilised in human and animal vaccines against a wide range of pathogens [4], [5], [20], this is to our knowledge the first assessment of a thermostabilised adenovirus-vectored vaccine in a vaccine target population. Further evaluation of the sugar-membrane thermostabilised ChAdOx1-GnGc vaccine in field efficacy studies involving large numbers of livestock in RVFV-endemic settings in Africa is clearly warranted. Such studies will allow a cost-benefit analysis of the manufacture, formulation and use of the thermostable product in comparison to the ‘cold chain’ ChAdOx1-GnGc vaccine and provide useful end-user data on its suitability for field deployment.

## Conflict of interest

The authors declare that they have no competing interests.
